# Whole-exome sequencing identifies novel pathogenic mutations and putative phenotype-influencing variants in Polish limb-girdle muscular dystrophy patients

**DOI:** 10.1186/s40246-018-0167-1

**Published:** 2018-07-03

**Authors:** Jakub Piotr Fichna, Anna Macias, Marcin Piechota, Michał Korostyński, Anna Potulska-Chromik, Maria Jolanta Redowicz, Cezary Zekanowski

**Affiliations:** 1 Department of Neurodegenerative Disorders, Mossakowski Medical Research Centre, Polish Academy of Sciences, 5 Pawinskiego St., 02-106 Warsaw, Poland; 2 Department of Neurology, Medical University of Warsaw, 1a Banacha St., 02-097 Warsaw, Poland; 3 Department of Molecular Neuropharmacology, Institute of Pharmacology, Polish Academy of Sciences, 31-344 Krakow, Poland; 4 Laboratory of Molecular Basis of Cell Motility, Department of Biochemistry, Nencki Institute of Experimental Biology, 3 Pasteur St., 02-093 Warsaw, Poland

**Keywords:** Limb-girdle muscular dystrophy, LGMD, Skeletal muscle, Exome, Next generation sequencing, NGS, WES

## Abstract

**Background:**

Limb girdle muscular dystrophies (LGMD) are a group of heterogeneous hereditary myopathies with similar clinical symptoms. Disease onset and progression are highly variable, with an elusive genetic background, and around 50% cases lacking molecular diagnosis.

**Methods:**

Whole exome sequencing (WES) was performed in 73 patients with clinically diagnosed LGMD. A filtering strategy aimed at identification of variants related to the disease included integrative analysis of WES data and human phenotype ontology (HPO) terms, analysis of genes expressed in muscle, analysis of the disease-associated interactome and copy number variants analysis.

**Results:**

Genetic diagnosis was possible in 68.5% of cases. On average, 36.3 rare variants in genes associated with various muscle diseases per patient were found that could relate to the clinical phenotype. The putative causative mutations were mostly in LGMD-associated genes, but also in genes not included in the current LGMD classification (*DMD*, *COL6A2*, and *COL6A3*). In three patients, mutations in two genes were suggested as the joint cause of the disease (*CAPN3*+*MYH7*, *COL6A3*+*CACNA1S*, *DYSF*+*MYH7*). Moreover, a variety of phenotype-influencing variants were postulated, including in patients with an identified already known primary pathogenic mutation.

**Conclusions:**

We hypothesize that LGMD could be better described as oligogenic disorders in which dominant clinical presentation can result from the combined effect of mutations in a set of genes. In this view, the inter- and intrafamilial variability could reflect a specific genetic background and the presence of sets of phenotype-influencing or co-causative mutations in genes that either interact with the known LGMD-associated genes or are a part of the same pathways or structures.

**Electronic supplementary material:**

The online version of this article (10.1186/s40246-018-0167-1) contains supplementary material, which is available to authorized users.

## Background

Limb girdle muscular dystrophies (LGMD) are a heterogeneous group of genetic disorders with similar clinical features, and a diverse and partially unknown genetic background. LGMD are characterized clinically by progressive muscle weakness and atrophy, predominantly or primarily of the pelvic and shoulder girdle muscles, without facial muscle dysfunction. The clinical course of the disease may be variable, ranging from severe forms with early onset and rapid progression to milder forms with later onset and minor physical disability. In the majority of cases, serum creatine kinase (CK) is elevated and a dystrophic pattern with necrosis and regeneration is observed on muscle biopsy [1, 2]. Most patients show a definable phenotype, but there are numerous exceptions as well as intrafamilial variability. LGMD are very rare disorders, fulfilling the criteria for orphan diseases with an estimated prevalence of 1/44000–1/123000 [3, 4].

Numerous studies performed world-wide in the last two decades have led to the identification of mutations in 30 genes (and one associated *locus*) causally involved in LGMD pathophysiology [5]. Current LGMD classification is based on the mode of inheritance and the particular gene involved. The two general categories, autosomal dominant LGMD1 and autosomal recessive LGMD2, are divided into subgroups with different alphabetic designators, each caused by mutations in a specific gene.

To date, eight LGMD1 and 23 LGMD2 subtypes have been recognized [6–8]. This list is still expanding, with seven subtypes added in the last 3 years. The diagnosis of muscular dystrophies (including within the LGMD group) is difficult due to the presence of a number of different conditions with similar clinical phenotypes, including limb-girdle muscle weakness, e.g., myofibrillar myopathies, Bethlem myopathy, Becker muscular dystrophy, facioscapulohumeral muscular dystrophy, and Emery-Dreifuss muscular dystrophy. In fact, some of the latter have been considered a form of LGMD [1, 6].

According to the latest guidelines, the precise diagnosis of LGMD should rely on a detailed clinical examination, muscle biopsy, and genetic analysis to detect the causative mutations [9]. However, muscle biopsy findings are often not sufficiently specific; therefore, genetic testing is considered the most reliable tool in LGMD diagnosis.

The molecular pathophysiology of LGMD is heterogeneous, with mechanisms ranging from defects in the dystrophin-dystroglycan complex, through abnormal localization of components of the muscle cytoskeleton and enzymatic defects, to sarcomeric and nuclear lamina dysfunctions. Different mutations in the same gene can cause widely different phenotypes (e.g., individual *FKRP* mutations cause a form of muscle-eye brain disease, a congenital severe muscular dystrophy, and a classical, adult LGMD form [10, 11]). The functional diversity of the protein products of the disease-causing genes makes the diagnosis very difficult and complex, requiring deep phenotyping.

It should be emphasized that despite intensive research, especially on the identification of novel causative genes, up to 50% of clinically defined LGMD cases are still without genetic diagnosis. Furthermore, the treatment of LGMD remains supportive and palliative, although it is expected that early diagnosis of the disease subtypes, based mainly on genetic testing, will allow the development of therapeutic strategies preventing or delaying the pathological process in the foreseeable future [12, 13]. Proactive multidisciplinary care and genetic counseling of LGMD patients is recommended, preferably immediately after diagnosis.

Since not all genetic risk factors of LGMD have been identified, further studies into the genetic background of the disease are necessary. Whole exome sequencing (WES) and, even more so, whole genome sequencing (WGS) provide a non-biased approach towards discovery of potentially causative mutations [14]. Subsequent comprehensive bioinformatic analyses of the resulting list of genomic variants could not only pinpoint novel genes that could be associated with the disease, but also reveal mutations in genes related to other disorders explaining some of the as-yet molecularly undiagnosed cases. Additionally, apart from the causative mutations, variants that could be called phenotype-influencing, or even co-causative, could add up to the clinical phenotype.

Here, we report genetic variants identified using WES and comprehensive bioinformatic analyses in a fairly large group of Polish patients with clinically diagnosed LGMD. We found putative pathogenic mutations in known myopathy-related genes in 68.5% of cases. In all the cases, we propose numerous possibly phenotype-influencing or even co-causative mutations, including in genes not previously related to LGMD.

## Methods

### Patients

The study involved 72 cases (73 patients including a pair of siblings) with clinically diagnosed limb-girdle muscular dystrophy from a single neuromuscular diagnosis and treatment medical center. LGMD was defined as a progressive muscle weakness and atrophy of the pelvic and shoulder girdle muscles, as well as proximal limb muscles, without an involvement of facial muscles. The diagnosis was made on the basis of clinical assessment and muscle biopsy. Childhood cases with early-onset asymptomatic persistent hyperCKemia were included when the muscle biopsy showed evident features of muscular dystrophy. Miyoshi myopathy phenotypes (muscular dystrophy with predominant calf atrophy and high CK level) were also included because of their considerable genetic and phenotypic overlap with LGMD2B and 2L. Other types of muscular dystrophies (Duchenne muscular dystrophy, Becker muscular dystrophy, facioscapulohumeral muscular dystrophy, Emery-Dreifuss muscular dystrophy, and myotonic dystrophy type 1 and 2) and other myopathies (congenital, metabolic, mitochondrial, myofibrillar, and inflammatory) were excluded on the base of their clinical, electrophysiological, and morphological characteristics. To avoid Becker muscular dystrophy cases, we included male patients who had either similarly affected female siblings or previous negative MLPA (multiplex ligation-dependent probe amplification) results (which excluded large deletions or duplications in the dystrophin gene) together with normal muscle immunofluorescence staining for dystrophin.

The mean age of the patients was 26 years (range 3–78). The basic clinical data are shown in a supplementary file (see Additional file 1). Thirty-nine probands had one or more affected siblings. Two patients (no. 243 and 407) had a positive family history suggesting autosomal dominant inheritance; however, no parent was available for clinical assessment. In four patients, there was a background of second-degree parental consanguinity.

We also performed WES for 12 patients with non-classic muscular disease phenotypes where an LGMD diagnosis could not be definitively excluded (see Additional file 1). Data from these cases, and from an additional 16 patients from seven families with non-muscular neurological diseases, were used for comparison during the bioinformatic assessment of WES results.

### Genetic analyses

DNA was extracted from peripheral blood using standard methods [15]. Whole exome sequencing (WES) was performed commercially at BGI Tech Solutions (Hong Kong) using a SureSelect Human All Exon v5+UTR enrichment kit and paired-end 100-nt sequencing on the Illumina HiSeq2000 platform. Fast read files were generated from the sequencing platform via the Illumina pipeline. Adapter sequences in the raw data were removed and low-quality reads with low base quality discarded. On average, 240,451,900 “clean” paired-end reads per sample were aligned to the human reference genome hg19 using the Burrows-Wheeler Alignment (BWA) package [16]. Duplicate reads were removed with Picard and base quality Phred scores were recalibrated using GATK’s covariance recalibration [17]. The obtained 15 Giga-bases of aligned sequence data resulted in 125x median coverage of the target capture regions with 97.4% of target bases covered at least 10×. Capture performance statistics were calculated using CollectHsMetrics in Picard 2.17.10. The alignments were viewed with an Integrative Genomics Viewer [18]. SNVs (single-nucleotide variants) and indels (small insertion/deletion) variants were called using the GATK Unified Genotyper. Annovar was used for initial variant annotation [19] with further annotation, filtering, and analysis performed on Galaxy (on PL-Grid Infrastructure) and GeneTraps (Intelliseq) platforms.

### Copy number variant analysis

Copy number variants (CNVs) were called using CODEX software (version 1.8) [20]. The analysis was performed within technical batches of samples. CNVs were annotated with allele frequencies using best-matching CNVs from 1000 genomes, and all the CNVs matching common CNVs (MAF > 1%) were removed. Genes overlapping each CNV in patients were identified using Ensembl version 86. The genes assigned to CNVs were annotated using diseases and phenotypes from Human Phenotype Ontology (HPO) [21] and tissue expression scores obtained from the GeneAtlas.

### Bioinformatic analyses

Whole exome sequencing identified on average 125,000 SNVs and 23,000 indels in each sample, of which 76,000 and 15,000, respectively, were off target (defined as intergenic or intronic, but not affecting splice sites) and therefore removed from further consideration. Only variants with an impact on coding regions were retained: missense, nonsense, frameshift, and essential splice site mutations. Further filtering was based on Phred quality scores, allele frequency in the ExAC (Exome Aggregation Consortium) database (< 3% for variants in genes already associated with LGMD, and < 1% for variants in other genes), association with HPO terms and predicted pathogenicity [22]. The HPO terms used were: “muscular_dystrophy,” “muscle_weakness,” “limb-girdle,” “myopathy,” “muscular_atrophy,” “muscle_atrophy,” and “creatine phosphokinase.” Variants predicted to be pathogenic by at least one of the following programs were taken into further consideration: Mutation Taster, PolyPhen2, and SIFT. In total, among all the samples, 1880 variants were analyzed further (see Additional file 2). Prioritization was based on the following: the predicted effect, with truncating and elongating variants being evaluated more carefully; the predicted pathogenicity; and, finally, known association with myopathic phenotypes. All these variants were individually assessed by a board of geneticists and clinicians according to the guidelines of the American College of Medical Genetics and Genomics [23], with emphasis on the actual phenotype of each patient. Variants were then categorized as putative pathogenic (fit the phenotype effect very well) or potentially phenotype-influencing (could be responsible for naturally occurring variability of symptoms in frame of a typical LGMD clinical phenotype), with other variants assessed as unlikely to be related to the disease.

Independently, additional analyses were carried out with Exomiser2, PhenIX and Exome walker, with prioritization of variants based on possible association with limb-girdle muscular dystrophy (HP:0006785), and (based) on random-walk analysis of protein interaction networks with proteins already associated with the LGMD phenotype [24].

**Fig. 1:**
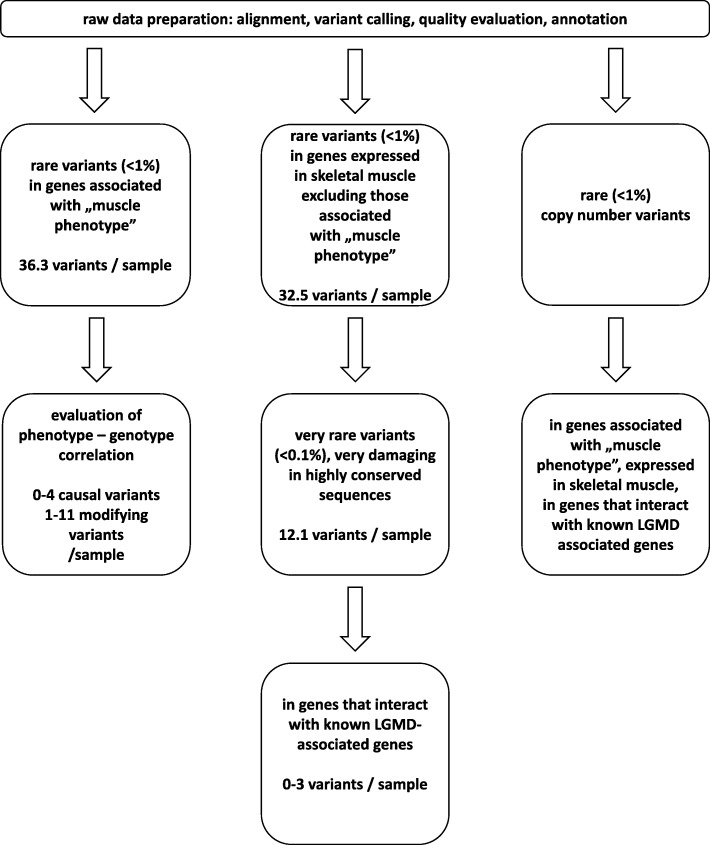
Whole exome sequencing analysis pipeline. Details of the methods are presented in the “Methods” section. Numbers of variants after each step of analysis are given

Additionally, in a second approach, rare (< 1% in ExAC database) variants in genes expressed in the human muscle, and in genes whose mouse homologs are expressed in muscle, were analyzed. Further analysis included ultra-rare variants (< 0.1% in ExAC), highly damaging mutations (including nonsense, frameshift, splice site mutations), interactome and association with pathways and structures that could play a role in LGMD pathogenesis. Known LGMD-associated genes were used to query the Biological General Repository for Interaction Datasets (BioGRID, version 3.4.151, accessed 3 August 2017) and Kyoto Encyclopedia of Genes and Genomes (KEGG, release 83.1, accessed 3 August 2017), which yielded potential interactors and muscle related pathways. Variants in genes that either interact with known LGMD-associated genes or are in the same pathway (as suggested by BioGRID and KEGG databases) were selected (see Additional file 3). Again, at the end of this discrimination pipeline (Fig. 1.), extracted variants were also correlated with patient phenotype and results of clinical examinations.

Selected variants (including all the putative causative mutations) were confirmed using direct fluorescence-based sequencing (ABI 3130 Genetic Analyzer, Applied Biosystems, USA). Segregation analysis including confirmation of trans configuration of compound heterozygotes could not always be performed because of the limited availability of DNA samples of the relatives.

## Results

Using whole exome sequencing, we found putative pathogenic mutations (*pathogenic* or *likely pathogenic* according to ACMG criteria) in known myopathy genes in 50 of the 72 LGMD cases (68.5%). In 43 cases, the identified variants were known to be pathogenic (found in OMIM, ClinVar, HGMD, or LOVD databases, or already described). These were associated mainly with the LGMD phenotype, but also with collagen-related myopathy and MYH-7 related myopathy. Putative causative mutations were found not only in LGMD-related genes (45 cases, 62.5%) but also in other myopathy-related genes (5 cases, 8%), highlighting the clinical overlap between muscular disorders. The latter cases had mutations in collagen myopathy-related genes (4 cases) and Becker’s muscular dystrophy (1 case). Selected variants most probably influencing the phenotype are presented in Table 1. A full version of the table including basic clinical data and evidence of pathogenicity is provided in a supplementary table (see Additional file 1).

**Table 1:** Putative causative mutations and genes with potentially phenotype-influencing variants identified by WES in 73 LGMD patients

Patient no.	Putative causative gene(s)	Genotype	Genes with mutations putatively influencing clinical phenotype
20	*ANO5*	p.D81G/p.R758C	*NEB*, *DES*, *TTN*
10	*ANO5*	p.D81G/p.W401X*	*BAG3*, *FLNC*, *CHRNE*, *CACNA1S*, *TTN x2*
173a	*CAPN3*	c.1193+1G>A (splice site)/c.598-612delGTTCTGGAGTGCTCT	*NEB x2*, *DNM2*, *TTN x2*, *CACNA1S*
424	*CAPN3*	c.598-612delGTTCTGGAGTGCTCT/p.G221S*	*COL12A1*, *PLEC*, *DNM2TTN*
186a	*CAPN3*	c.550delA/p.A609E	*LDB3/ZASP x2*, *COL6A2*, *COL6A3*, *SGCD*, *POMT1*, *DYSF*, *SYNE2*, *MYH6*, *B3GALNT2*
175d	*CAPN3*	c.550delA/c.598-612delGTTCTGGAGTGCTCT	*MYOT*, *SGCB*, *RYR1*, *NEB*, *SYNE2*, *TTN*
12	*CAPN3*	c.550delA/c.550delA	*COL6A3*, *FLNC*, *NEB*,*TTN x2*
144	*CAPN3*	c.550delA/c.550delA	*DNM2*, *TMEM5*, *TTN x2*
212	*CAPN3*	c.550delA/c.550delA	*DYSF*, *TTN x6*
127	*CAPN3*	c.550delA/c.550delA	*RYR1*, *FLNC*, *SYNE2*, *TTN x4*
184a	*CAPN3*	c.550delA/c.550delA	*HSPG2*, *TTN*
6	*CAPN3*	c.550delA/c.550delA	*TRAPPC11*, *RYR1*, *LAMA2*, *FLNC*, *NEB*, *PPARGC*, *TTN*, *MYF6*
764	*CAPN3*	c.550delA/c.1722delC	*LDB3/ZASP x2*, *POMT1*, *TMEM43*
18	*CAPN3* *MYH7*	c.550delA/p.E566K p.R204H	*LDB3/ZASP x2*, *GBE1*, *TTN x4*,
TO	*CAPN3*	c.550delA/p.G221S*	*TRAPPC11*, *LIPE*, *GBE1*, *HSPG2*, *TTN x3*
8	*CAPN3*	c.550delA/p.P82L	*NEB x2*, *COL6A3*, *SYNE1 x2*, *TTN x5*, *LDB3/ZASP*, *HSPG2*
13	*CAPN3*	c.550delA/p.R147X	*COL12A1*, *NEB*
4	*CAPN3*	c.550delA/p.R355W	*FLNC*, *SYNE1*, *DCTN1*, *TTN x3*
433	*CAPN3*	c.550delA/p.R448C	*COL6A3*, *TARDBP*, *TTN x2*
668	*CAPN3*	c.550delA/p.T560A	*PLEC x 3*, *SYNE1 x2*, *CCDC78*, *COL9A3*, *HSPG2*
193a	*CAPN3*	c.550delA/p.W130R*	*COL6A3*, *NEB*, *HSPG2*, *TTN x2*, *GNE*
113	*CAPN3*	p.R748X/c.1722delC	*COL6A3*, *RYR1*, *HSPG2*, *SYNE1 x2*, *DCTN1*, *TTN x2*
144a	*CAPN3*	p.R748X/c.598-612delGTTCTGGAGTGCTCT	*COL6A1*, *COL6A3*, *HNRNPDL*, *RYR1 x2*, *SYNE1*, *MYH7*, *TTN x5*
225	*CAPN3*	p.P102L/p.S606L	*MYH3*, *SYNE1*, *SYNE2*, *TTN*
196	*COL6A2*	p.G277E*	*CAV3*, *LAMA2*, *ANO5 – ITGA7*, *RYR1*, *SYNE2*, *TTN x3*
901	*COL6A3* *CACNA1S*	p.E1386K/p.R2420W p.T349I*	*NEB*, *TTN*
7	*COL6A3*	p.R2142X*/p.K2483E	*FLNC*
275	*COL6A3*	p.T1368M/p.V2398I	*DAG1*, *NEB*, *SYNE1*, *TTN*
135	*DMD*	c.678-681delCTT*	*RYR1*, *ITGA7*, *DYSF*, *CCDC78*, *COL9A3*
275B	*DNAJB6*	p.G77E	*COL6A2*, *DAG1*, *DYSF*, *ISPD*, *NEB*, *RYR1*, *SYNE1*, *CHRNE*, *TTN x3*
192	*DYSF*	c.4821delG*/c.5058-1G>T* (splice site)	*LDB3/ZASP*, *ANO5*, *PLEC*, *SYNE1*, *TTN*
16	*DYSF*	p.D1876N/p.D1876N / c.5179delA*/c.5179delA*	*FLNC*, *DMD*, *MYH6*, *COL9A3*, *NIPA1*, *HSPG2*, *TTN x2*
219	*DYSF*	p.D1876N/p.E1763D/c.5179del*A	*PLEC x2*, *LDB3/ZASP x2*, *COL6A2*, *FKRP*, *COL12A1*, *TTN x3*
24 (family A)	*DYSF*	p.Q1323E/c.5237delG*	*COL6A3*, *MYH3*, *LDB3/ZASP*
3 (family A)	*DYSF*	p.Q1323E/c.5237delG*	*PLEC*, *COL6A3*
407	*DYSF* *MYH7*	p.V374L/c.5946G>A (splice site) p.A1487T	*ANO5*, *NEB*
15	*FKRP*	p.L276I/c.253+2T>C (splice site)	*PLEC x2*, *LARGE*, *KBTBD13*, *DCTN1*, *MYPN*, *TTN x2*
198	*FKRP*	p.L276I/c.650-667del CGCCCGCTATGTGGTGGG*	*COL6A3*, *COL4A1*, *NEB x2*, *TTN*
KW	*FKRP*	p.L276I/p.L276I	*ISPD*, *DYSF*, *ITGA7*, *SYNE1*, *TTN x2*
5	*FKRP*	p.L276I/p.L276I	*PLEC x2*, *COL6A3 x2*, *DYSF*, *POMGNT2*, *FLNC*, *TTN x3*
102	*FKRP*	p.L276I/p.L276I	*COL12A1*, *MYH2*, *SYNE2*, *TTN*
84e	*FKRP*	p.L276I/p.P217Q*	*TCAP*, *COL6A2*, *TTN x2*
CM	*FKRP*	p.L93P/p.R270C	*CAPN3*, *DMD*, *NEB*, *SYNE1 x2*, *CCDC78*, *TTN x4*
19	*LMNA*	p.G523R	*CAPN3*, *COL6A3*, *PLEC x3*, *RYR1*, *HSPG2*, *SYNE1*, *MYH3*, *LMOD3*, *RBM20*, *TNNI3K*
21	*SGCA*	p.V247M/p.V250L* (splice site)	*COL6A1*, *COL6A2*, *MYH2*, *LDB3/ZASP*, *POMT1*
84a	*SGCA*	p.V250L* (splice site) / p.R284C	*LDB3/ZASP x2*, *RYR1*, *COL6A2*, *COL6A3*, *SYNE1*
157	*SGCB*	p.S114F/p.I119N*	*PLEC x2*, *TRAPPC11*, *HSPG2 x2*, *TTN*
201	*SGCB*	p.S114F/p.S114F	*PLEC x2*, *TRAPPC11*, *B3GALNT2*, *HSPG2*, *SYNE1*
270a	*TCAP*	c.358-359delGA*/c.358-359delGA*	*NEB X3*, *SYNE1*, *BVES*, *TTN x2*
229a	*TRAPPC11*	p.D26G*/p.D26G*	*NEB*, *ITGA7*, *POMGNT1*
448a			*CAPN3*, *TTN (likely pathogenic fs)*,
179			*CAPN3*, *COL6A2*, *DNM2*, *-BVES*, *TTN x4*
214			*CAPN3*, *COL6A3*, *POMT2*, *COL12A1*, *TTN x2*
191			*CAPN3*, *FKRP*, *TTN x3*
658			*CAPN3*,, *MYPN*, *TARDBP*, *TTN x2*
752			*CAPN3*, *POMT2*, *FLNC x5*, *NEB*, *HSPG2*, *SYNE2*, *TTN*
170			*CAPN3*, *SGCA*, *RYR1*, *CACNA1S*, *LDB3/ZASP*,
250a			*CAPN3*, *SGCD*, *HSPG2*, *TTN*
130a			*CAPN3*, *PLEC x2*, *SYNE1 x2*, *SYNE2*, *CACNA1S*, *TTN*
160a			*CAPN3*, *BAG3*, *DES*, *NEB x2*, *TTN x2*, *CACNA1S*
128a			*RYR1 x2*, *COL6A3*
243			*BVES x2*, *SYNE1*, *TTN*, *HSPG2*, *HACD1*
592			*BAG3*, *TMEM43*, *TTN x3*, *HSPG2*
197			*COL6A3*, *ANO5*, *NEB*, *COL12A1*, *MYH3*, *SYNE1*, *TTN x2*, *SCN4A*, *LMNB2*
17			*DMD*, *PLEC x2*, *LAMA2*, *ITGA7*, *MYH6*, *SYNE2*, *CACNA1S*, *NEB*
195			*DNM2*, *TRIM32*, *POMGNT1*, *FLNC*, *NEB*,
14			*FLNC x2*, *TTN x2*
1038			*DYSF*, *PLEC*, *SYNE1*, *SYNE2*, *TTN x2*
9			*RYR1 x2*, *NEB*, *MYH7*, *FLNC*, *TTNx2*
155			*RYR1*, *ISPD*, *POMGNT2*, *COL6A2 DYSF*, *NEB*, *MYH3*, *TTN x3*
11			*TRAPPC11*, *NEB*, *HSPG2*
194			*HSPG2*, *TTN*
859			*CACNA1S*

*Indicates novel variants; RefSeq transcript reference sequences as in the LOVD database: ANO5 - NM_213599.2, CACNA1S - NM_000069.2, CAPN3 - NM_000070.2, COL6A2 - NM_001849.3, COL6A3 - NM_004369.3, DMD - NM_004006.2, DNAJB6 - NM_058246.3, DYSF - NM_003494.3, FKRP - NM_024301.4, LMNA - NM_170707.3, MYH7 - NM_000257.2, SGCA - NM_000023.2, SGCB - NM_000232.4, TCAP - NM_003673.3, TRAPPC11 - NM_021942.5

The dominant forms of LGMD were relatively uncommon, with only one case each of LGMD1B (patient19) and LGMD1E (patient 275B). In two cases, mutations in *MYOT* (patient 175d) and *CAV3* (patient 196) could also be considered responsible for the disease, but mutations in other genes (*CAPN3* and *COL6A2*, respectively) were assessed as better explaining the patients’ phenotypes. It is noteworthy that all four mutations, G523R in *LMNA*, G77E in *DNAJB6*, R126H in *CAV3*, and R370C in *MYOT*, are ultra-rare, with a prevalence of < 0.02% in the European population according to the ExAC database.

In 22 cases, we found homozygous or compound heterozygous mutations in *CAPN3*. Additionally, in 13 cases, we found only a single heterozygous putative pathogenic mutation in *CAPN3*, and 11 of these cases were in the group of patients without an identified causative mutation. These cases were familial and clearly autosomal recessive.

Mutations in *DYSF* (homozygous or compound heterozygous) were found to be responsible for the disease in six patients from five families. Interestingly, we found the same compound heterozygous mutations in the *DYSF* gene in siblings with discordant phenotypes (LGMD in the sister, Miyoshi myopathy in the brother). In seven cases, we found mutations in *FKRP*; sarcoglycanopathies were represented by four families: two cases with mutations in *SGCA* and two in *SGCB*. Another two cases had *ANO5* mutations.

In five cases, we found putative causative mutations in genes typically associated with other forms of myopathy. Four families had mutations in *COL6A2* and *COL6A3*, typically associated with Bethlem myopathy, but recently also found in LGMD-like cases. In one male proband, a single known pathogenic deletion of three nucleotides was found in the *DMD* gene.

In all the cases, additional variants that could relate to case-specific muscle weakness phenotypes were found. Possible causes of phenotypic overlap, and of inter- and intrafamilial (patients 24 and 3) differences, were mutations in other LGMD-associated genes, and mutations in genes associated with myofibrillar myopathy, congenital muscular dystrophy, collagen myopathy, Duchenne / Becker muscular dystrophy, Emery-Dreifuss muscular dystrophy, or cardiomyopathy. A number of other variants in muscle pathogenesis-related genes were found, with uncertain significance. Filtering of the variants annotated with myopathy phenotype-related HPO terms returned between 21 and 59 variants per sample, with an average of 36.3 per sample.

Analysis of genes expressed in muscle (based on the Geneatlas database) gave 2036 variants (total for all analyzed patients), of which 1271 were ultra-rare (< 0.1%) and 214 had a putative high impact on the protein (nonsense, frameshift, splice site mutations). Interactome-based analysis of these variants reduced their number to 83 in 20 genes associated with known LGMD-related genes (Table 2). A supplementary table lists variants found in genes whose products interact with myopathy-related proteins (see Additional file 3).

**Table 2:** Genes expressed in muscle and components of the interactomes of known LGMD genes

Gene	Protein	Interactive partner
*ANK1*	ankyrin 1	*RYR1*, *TTN*
*ANKRD23*	ankyrin repeat domain 23	*TTN*
*ATP1B4*	ATPase beta 4 polypeptide	*POMT1*, *POMT2*
*C1QTNF9*	C1q and tumor necrosis factor protein 9	*COL6A1*, *COL6A2*
*C1QTNF9*	C1q and tumor necrosis factor protein 9B	*COL6A1*, *COL6A2*
*EVC2*	Ellis-van Creveld syndrome 2	*TOR1AIP1*
*FYCO1*	FYVE and coiled-coil containing 1	*LMNA*
*HECW2*	HECT, C2 and WW containing E3 ubiquitin	*DYSF*
*HSPB2*	heat shock protein 2	*BAG3*, *CRYAB*, *FLNC*, *TCAP*, *TTN*
*MLIP*	muscular LMNA-interacting protein	*LMNA*
*MYOZ1*	myozenin 1	*FLNC*, *TCAP*
*MYOZ2*	myozenin 2	*FLNC*, *TCAP*
*MYOZ3*	myozenin 3	*FLNC*, *TCAP*
*OPRM1*	opioid receptor mu 1	*TNPO3*
*PDLIM7*	PDZ and LIM domain 7	*BAG3*, *PLEC*
*RXRA*	retinoid x receptor alpha	*TRIM32*
*SIRT2*	sirtuin 2	*DMD*, *DNAJB6*
*SRRM2*	serine/arginine repetitive matrix 2	*LMNA*, *PLEC*
*SVIL*	Supervillin	*LMNA*
*TRIM63*	tripartite motif containing 63, E3 ubiquitin protein ligase	*DES*, *FLNC*, *MYOT*, *TCAP*, *TTN*

Rare copy number variants in LGMD-related genes were found in 18 cases. A supplementary file shows detected CNVs in detail, not only in LGMD-related genes but also in the interactome of those genes, in other myopathy-related genes, and in genes expressed in muscles (see Additional file 4). It must be stressed that CNV predictions from WES data are not completely reliable [25, 26] and are presented in the supplementary material for indicative purposes only.

## Discussion

We performed the first comprehensive genetic analysis of patients with clinically defined LGMD in the Polish population.

On average, 36.3 rare variants per sample possibly related to the myopathic phenotype were identified. These variants were located in genes previously implicated in diverse muscle diseases (not just LGMD). These genes can be grouped according to the functional or structural association of their products: (i) dystrophin glycoprotein complex (*SGCA*, *SGCB*, *SGCD*, *SGCG*, *DAG1*), (ii) sarcomere structure (*TCAP*, *TTN*, *PLEC*, *DES*, *MYOT*) or assembly (*CAPN3*, *DNAJB6*), (iii) glycosylation (*FKRP*, *POMT1*, *POMT2*, *POMGNT1*, *ISPD*), (iv) signal transduction (*CAV3*, *DAG1*, *BVES*), (v) trafficking (*TRAPPC11*, *CAV3*, *DYSF*, *BVES*), and (vi) splicing (*TNPO3*, *HNRPDL*). After confirming the consistency of these results with the clinical and pathological characteristics of the patients, highly probable pathogenic genotypes could be identified in 50 out of 72 cases (68.5%). The above results gave a similar diagnostic rate to other recent NGS (next-generation sequencing) studies in genetically undiagnosed cohorts of LGMD: 47% in the Czech Republic, 62% in China, and 76% in Saudi Arabia [27–29]. Lower yields have been reported in studies involving patients pre-screened by targeted gene sequencing: 33% in Germany, 40% in the USA, and 45% in Australia [30–32]. The distribution of LGMD subtypes was similar to those observed in Germany [30] and Italy [33], with *CAPN3* being the most frequent main putative pathogenic cause, and frequent cases with *FKRP* and *DYSF* mutations.

It should be noted that putative causative mutations in genes not included in the LGMD classification, *COL6A2*, *COL6A3*, and *DMD*, have also been reported by other authors in their LGMD cohorts [27–31], indicating phenotypic overlap between LGMD and other myopathies, making clinical diagnosis difficult in some cases. As a result of WES analysis, a diagnosis correction was made in the case of patient 135 to Becker muscular dystrophy. In the case of patients with causative mutations in *COL6* genes, the diagnosis was also changed to suspected collagen myopathy (as *COL6* genes mutations are not included in the current LGMD classification). In other cases, including those that were genetically unsolved, we upheld the clinical diagnosis of LGMD.

In the unsolved cases, the pathogenic mutations or copy number variants could be located in noncoding, regulatory or deep intronic regions. This could explain, for instance, the excess of single *CAPN3* mutation carriers as compared to population-wide data. It is also likely that additional co-responsible *CAPN3* mutations are located in the regulatory regions of the gene and therefore missed by exome sequencing. In sporadic or first cases in a family, a post-zygotic mutation event in the muscle could also be the cause of the disease [34].

In at least three cases, the patient’s clinical phenotype could be plausibly explained by mutations in more than one gene (*CAPN3* + *MYH7*, *COL6A3* + *CACNA1S*, *DYSF* + *MYH7*). In these cases apart from clinical phenotype of LGMD, additional features included the following: considerable distal weakness with early onset typical for MYH7-related myopathies (patient 18, *CAPN3* + *MYH7*), early disease onset not typical for LGMD2B and possible autosomal dominant inheritance (patient 407, *DYSF* + *MYH7*), and almost exclusively type 1 fibers in biopsy unexpected for LGMD or Bethlem myopathy, whereas encountered in CACNA1S-related myopathies (patient 901, *COL6A3* + *CACNA1S*). In these cases, a clinical diagnosis was upheld with possible co-existing MYH7 and CACNA1S-related myopathy. Likewise, in the majority of cases, additional variants in other genes apart from the highly probable major pathogenic mutations could at least add to the phenotypic manifestation; however, selecting co-causative variants from those classified as potentially modifying was not possible. Discrimination between possible phenotype-influencing variants and thousands of insignificant variants harbored by each individual became one of the most difficult novel challenges. Indeed, in all the studied cases, we encountered novel and rare variants related to LGMD and other myopathies, but their relevance could not be established based on the inheritance mode, patient’s phenotype, and known effect of mutations in these genes.

Here, we adopted a strategy for identifying the phenotype-influencing variants that linked the genes bearing found variants with any of the terms from the HPO database pointing to muscle physiology or structures. However, this approach could result in missing variants located in genes not yet associated with muscle disease, or missing variants coding for an interactome of the known causative proteins. Therefore, we additionally tried to identify putative phenotype-influencing variants by comprehensively analyzing those with MAF < 0.1%, expressed in muscle (human and/or murine) and markedly influencing the structure/function of the encoded protein (human and/or murine), but with no known association with the myopathy clinical phenotype (therefore excluding variants identified in the first approach). These variants were analyzed further based on the association of respective genes with known LGMD-related genes or pathways in which LGMD-related genes are involved. This reduced the overall number of such variants to 68 in 19 genes (0–3 per case). In light of their inheritance pattern, their presence in our in-house WES/WGS control group as well, and the presence of other variants that seem to explain the phenotype well in many cases, it is unlikely that the aforementioned 68 variants are causative. Still, putative phenotype-influencing variants could be within those in genes expressed in skeletal muscle.

By using various filtering approaches to WES results, one can gain insight into the possible influence of new genes on the disease. A list of such selected genes previously not associated with LGMD, but, according to our analysis, with a likely effect on the disease, is presented in Table 3.

**Table 3:** Selected genes with reported skeletal muscle expression which could contribute to LGMD

Gene	Protein	Interacts with
*OBSCN*	Obscurin	*TTN*
*MAP4*	microtubule-associated protein 4	*BAG3*, *TARDBP*
*MAST2*	Microtubule-associated serine/threonine kinase 2	*DMD*
*CACNA1S*	calcium channel, voltage-dependent, L type, alpha 1S subunit	–
*MYH7*	myosin heavy chain 7	*TPM2*

All the genes listed in Table 3 have already been examined in the context of muscular disorders, as well as muscle structure and functioning (see Additional file 5).

Numerous genetic muscular disorders phenotypically overlap with LGMD, as limb-girdle weakness is one of their common symptoms. NGS-based genetic analyses can resolve clinical dilemmas and facilitate exact diagnosis [35–37]. Additionally, with the reporting of new cases, the spectrum of clinical manifestations of mutations in a given gene is likely to expand. Moreover, recent mass sequencing results show that the genetic background is more complex than previously considered [5, 6]. Also, our data suggest that mutations in more than one gene in a single patient can result in the LGMD phenotype. Taking into account the phenotypic variability within a given LGMD subtype or even between patients with the same causative mutation [38–40], one should expect a strong influence of disease-modifying genes, although no specific modifier or co-causative genes have been described to date. In our patients with identified primary causative mutations, at least a dozen additional variants that could influence or modify the phenotype were found, even when only genes known to be associated with muscle pathology were taken into consideration. It is therefore likely that the spectrum of genetic factors influencing the disease is substantially wider than previously recognized.

Indeed, a common polymorphism in the *LTPB4* gene has been shown to be a disease-modifying factor in dystrophinopathy [41]. Moreover, in some cases, mutations in more than one gene could be necessary to cause the disease [42]. Thus, digenic inheritance has been proven for a subtype of facioscapulohumeral muscular dystrophy [43] found in congenital myasthenic syndrome [44], and it has also been suggested for calpainopathy [45].

Proteins of the muscle cell form a complex machinery where structural or functional impairment of any of its components can result in progressive muscle dysfunction and eventual destruction. The mutational burden in the numerous genes involved in muscular diseases must not be overlooked, as accumulation of minor defects, even those without an apparent overall effect when present in isolation, could result in a similar phenotype. Indeed, oligogenic etiology may be most easily observable in unsolved, sporadic LGMD cases, where the main putative causative mutation has not been identified. Interestingly, two of our “double trouble” cases were found precisely in sporadic patients.

On the other hand, some of the mutations described as disease-causing prior to the NGS era might only have a modifying effect, incomplete penetrance, and require additional variants to bring about pathology [40, 46]. The overrepresentation of single heterozygous CAPN3 mutations in our group may also indicate digenic or oligogenic inheritance.

Multiple annotation tools have become available using various algorithms and databases to predict the functional effects of genomic variants. One should bear in mind, however, that the functional scores of a given variant may differ substantially between different databases and prediction tools as they can be based on different functional aspects and prior knowledge. The superiority of high-scale bioinformatic analysis over focused genetic studies lies in the possibility of repeating the analysis and making use of novel knowledge [47].

Ideally, genetic testing should be combined not only with deep phenotyping but also with comprehensive analyses of transcripts and protein isoforms to pinpoint novel causative, co-causative, and phenotype-modifying variants.

## Conclusions

The availability of exome and whole genome data for various conditions, including LGMD, challenges the classical definition of genetic causality and the concept of strictly monogenic disorders [48] and underlines the heterogeneity and complexity of the human genome [49]. Our results show a range of phenotypes associated not only with genes previously and typically associated with LGMD but also with genes related to similar muscular disorders, such as Bethlem myopathy, myofibrillar myopathy, or congenital muscle dystrophy, as well as with genes not previously considered in the context of myopathies. Even if it is not always possible to prove the effect of putative modifying variants on the phenotype, aggregate analysis of mutations suggests that the sheer “variant burden” contributes to phenotypic variability.

Based on the obtained exomic data, we propose that LGMD could be better defined as a group of oligogenic disorders, in which variable clinical symptoms result from the combined effects of mutations in a set of genes and can result in a broad spectrum of clinical presentation rather than distinct disease entities. This could explain the fact that NGS methods fail to identify a single main causative gene in many LGMD cases, but indicate a range of possibly pathogenic and/or co-causative mutations in almost every case.

This could also explain the clinical heterogeneity not only of LGMD or within subtypes but also among individuals harboring the same known pathogenic mutations, and even between affected members of the same family. While a considerable proportion of LGMD cases can be easily attributed clinically to a single gene, the high number of variants that could relate to myopathy and sometimes to specific phenotype features in cases with mutations in known LGMD-associated genes suggests that the oligogenic nature of the disease may be important even in patients with a well-defined primary pathogenic cause.

However, unequivocal identification of such modifying variants requires comprehensive bioinformatic analyses integrated with deep phenotyping to make a final diagnosis [50]. It should be remembered, nevertheless, that it is practically impossible to ascertain the causality even of a single gene in a single subject or a risk-family [51]. Identification of all risk or co-causative factors requires bioinformatical analysis of combined genomic and clinical data on large groups of ethnically diverse patients with various muscle diseases followed by functional in vitro studies.

We expect that with the appearance of genomic data from large groups of patients with a large spectrum of myopathies, it will become possible to examine not just a limited number of genes and variants, but groups of genes encoding entire pathways and modules [52]. As a result, the traditional descriptive classification of muscle diseases will transform into a systemic and pathway-based view of clinical phenotypes [53]. The presented results are the first and indispensable step towards this goal of translational medicine.

## Additional files

Additional file 1: Clinical characteristics and WES results (including evidence of pathogenicity according to ACMG criteria) of 85 patients studied. (XLSX 24 kb)

Additional file 2: Lists of rare (< 1%) variants in genes related with “muscle phenotype” for individual patients. (XLSX 3003 kb)

Additional file 3: List of variants in genes expressed in the human muscle and in genes whose mouse homologs are expressed in muscle, which are in the interactome of known LGMD- and MFM-associated genes. (XLSX 1451 kb)

Additional file 4: List of copy number variants (CNVs) identified in the vicinity of LGMD-related genes. (XLS 2184 kb)

Additional file 5: Selected genes with reported skeletal muscle expression which could contribute to LGMD. (DOCX 16 kb)

## Abbreviations

BWA: Burrows-Wheeler alignment (software)
CK: Serum creatine kinase
CNV: Copy number variant
ExAC: The exome aggregation consortium (database)
GATK: Genome analysis toolkit (software)
HGMD: Human gene mutation database
HPO: The human phenotype ontology (database)
indel: Small insertion/deletion
LGMD: Limb-girdle muscular dystrophy
LOVD: Leiden open variation database
MLPA: Multiplex ligation-dependent probe amplification
NGS: Next-generation sequencing
OMIM: Online Mendelian inheritance in man (database)
PCR: Polymerase chain reaction
SIFT: Sorting intolerant from tolerant (software)
SNV: Single-nucleotide variation
WES: Whole exome sequencing
WGS: Whole genome sequencing
