# Supplementary material for: Whole-exome sequencing identifies novel pathogenic mutations and putative phenotype-influencing variants in Polish limb-girdle muscular dystrophy patients
Source: Hum Genomics. 2018 Jul 3;12:34. doi: 10.1186/s40246-018-0167-1 (PMC6029161; doi:10.1186/s40246-018-0167-1)
Supplement: Supplementary file 5 — Selected genes with reported skeletal muscle expression which could contribute to LGMD. (DOCX 16 kb) [file 40246_2018_167_MOESM5_ESM.docx]

By using various filtering approaches to WES results one can gain insight into possible influence of new genes on the disease. A list of such selected genes previously not associated with LGMD, but, accordingly to our analysis, with a likely effect on the disease is presented in the table below.

Table. Selected genes with reported skeletal muscle expression which could contribute to LGMD

| Gene | Protein | interacts with |
| --- | --- | --- |
| *OBSCN* | Obscurin | *TTN* |
| *MAP4* | microtubule-associated protein 4 | *BAG3, TARDBP* |
| *MAST2* | microtubule associated serine/threonine kinase 2 | *DMD* |
| *CACNA1S* | calcium channel, voltage-dependent,  L type, alpha 1S subunit | - |
| *MYH7* | myosin heavy chain 7 | *TPM2* |

All the genes listed in Table 4 have already been examined in the context of context of muscular disorders, as well as muscle structure and functioning. For instance, *OBSCN* mutations were linked with cardiomyopathy, and a possible contribution to muscular dystrophy was also discussed, since it was known that LGMD-associated titin (*TTN*) mutations weaken or abrogate its binding to obscurin.[1] In the presented study we found 25 rare (<1%) mutations in *OBSCN,* including two frameshift and a nonsense one in 26 patients. In eight cases two or three heterozygous mutations were present.

The role of *MAP4* in the microtubule network and its possible involvement in muscle disease has also been considered.[2,3] We found eight rare single heterozygous *MAP4* mutations in ten patients.

Microtubule Associated Serine/Threonine Kinase 2 encoded by *MAST* is a postulated link between the dystrophin /utrophin (*DMD*/*UTR*) network and microtubule filaments [4] and, together with *OBSCN,* was already indicated as a candidate gene in myopathy in other next generation sequencing studies.[5,6] We found eight rare single heterozygous *MAST* mutations in eight patients.

*CACNA1S* mutations were described in hypokalemic periodic paralysis and malignant hyperthermia, but recently they were also found in several cases of autosomal recessive or dominant congenital myopathy.[7] Rare *CACNA1S* mutations were found in eight of our patients, and in one of them they were deemed highly responsible for the phenotype involving almost exclusively type I fibers in the biopsy.

*MYH7* mutations were described in cardiomyopathy but also in cases of congenital myopathy without cardiac manifestations.[8] We found rare *MYH7* mutations in four patients. In two cases specific features including early onset and footdrop were characteristic for *MYH7* myopathy.

1. Fukuzawa A, Lange S, Holt M, Vihola A, Carmignac V, Ferreiro A et al. Interactions with titin and myomesin target obscurin and obscurin-like 1 to the M-band: implications for hereditary myopathies. J Cell Sci. 2008;1;121(11):1841-51 doi: 10.1242/jcs.028019.
2. Mogessie B, Roth D, Rahil Z, Straube A. A novel isoform of MAP4 organises the paraxial microtubule array required for muscle cell differentiation. [Elife.](https://www.ncbi.nlm.nih.gov/pubmed/?term=A+novel+isoform+of+MAP4+organises+the+paraxial) 2015 Apr 21;4:e05697. doi: 10.7554/eLife.05697.
3. Chinnakkannu P, Samanna V, Cheng G, Ablonczy Z, Baicu CF, Bethard JR, et al. Site-specific microtubule-associated protein 4 dephosphorylation causes microtubule network densification in pressure overload cardiac hypertrophy. J Biol Chem. 2010;9;285(28):21837-48. doi: 10.1074/jbc.M110.120709.
4. Liu W, Wu A, Pellegrini M, Wang X. Integrative analysis of human protein, function and disease networks. Sci Rep. 2015;24;5:14344. doi: 10.1038/srep14344.
5. Abath Neto O, Tassy O, Biancalana V, Zanoteli E, Pourquié O, Laporte J. Integrative data mining highlights candidate genes for monogenic myopathies. PLoS One. 2014;29;9(10):e110888. doi: 10.1371/journal.pone.0110888.
6. Pedrotti S, Giudice J, Dagnino-Acosta A, Knoblauch M, Singh RK, Hanna A et al. The RNA-binding protein Rbfox1 regulates splicing required for skeletal muscle structure and function. Hum Mol Genet. 2015;15;24(8):2360-74. doi: 10.1093/hmg/ddv003.
7. Schartner V, Romero NB, Donkervoort S, Treves S, Munot P, Pierson TM et al. Dihydropyridine receptor (DHPR, CACNA1S) congenital myopathy. Acta Neuropathol. 2017;133(4):517-533. doi: 10.1007/s00401-016-1656-8.
8. Fiorillo C, Astrea G, Savarese M, Cassandrini D, Brisca G, Trucco F et al. MYH7-related myopathies: clinical, histopathological and imaging findings in a cohort of Italian patients. Orphanet J Rare Dis. 2016;7;11(1):91. doi: 10.1186/s13023-016-0476-1.
